# Age-Dependent Male Mating Investment in *Drosophila pseudoobscura*

**DOI:** 10.1371/journal.pone.0088700

**Published:** 2014-02-19

**Authors:** Sumit Dhole, Karin S. Pfennig

**Affiliations:** Department of Biology, University of North Carolina, Chapel Hill, North Carolina, United States of America; CNRS, Université de Bourgogne, France

## Abstract

Male mating investment can strongly influence fitness gained from a mating. Yet, male mating investment often changes with age. Life history theory predicts that mating investment should increase with age, and males should become less discriminatory about their mate as they age. Understanding age-dependent changes in male behavior and their effects on fitness is important for understanding how selection acts in age-structured populations. Although the independent effects of male or female age have been studied in many species, how these interact to influence male mating investment and fitness is less well understood. We mated *Drosophila pseudoobscura* males of five different age classes (4-, 8-, 11-, 15-, 19-day old) to either young (4-day) or old (11-day) females, and measured copulation duration and early post-mating fecundity. Along with their independent effects, we found a strong interaction between the effects of male and female ages on male mating investment and fitness from individual matings. Male mating investment increased with male age, but this increase was more prominent in matings with young females. Male *D. pseudoobscura* made smaller investments when mating with old females. The level of such discrimination based on female age, however, also changed with male age. Intermediate aged males were most discriminatory, while the youngest and the oldest males did not discriminate between females of different ages. We also found that larger male mating investments resulted in higher fitness payoffs. Our results show that male and female ages interact to form a complex pattern of age-specific male mating investment and fitness.

## Introduction

Male reproductive success is generally limited by the number of available mates [1]. Consequently, males are expected to mate indiscriminately so as to maximize the number of matings they obtain [2], [3]. If, however, mating involves high costs for males (e.g. due to expensive ejaculates), males may discriminate between potential mates by preferring, or providing greater investment in, fitness-enhancing mates [3], [4], [5], [6], [7]. This discrimination by males is often expressed as facultative adjustments in their mating investment depending on female size, age, or fecundity [8], [9], [10], [11], [12], [13], [14], [15], [16], [17]. Such context-dependent alteration of mating investment can also depend on the risk of competition with other males, especially the likelihood of sperm competition [7], [18], [19], [20], [21], [22].

Context-dependent alteration of mating investment can be beneficial for males only if they are likely to mate again [23], [24]. Yet, the chances of future mating opportunities generally decrease with age. Life history theory therefore predicts that males should invest more resources in current matings, and should become less discriminatory as they age [23], [24], [25], [26], [27], [28].

In the same way that a male’s age may affect his investment in a given mating, a female’s age may also affect her reproductive effort. As females age, they too are expected to invest more of their remaining resources into a given reproductive event as opportunities for future reproduction diminish [25], [26], [27], [28]. However, because females are often resource limited, the relationship between female age and offspring production is not necessarily straightforward. Indeed, multiple studies have shown that female fecundity generally decreases with age due to senescence (for examples, see [29], [30]). Regardless of whether female fecundity increases or decreases with female age, any such relationship will generate an effect of female age on her mate’s fitness. Consequently, female age is a key feature that might affect male mating investment (for examples, see [14], [15, [31], [32]).

Conversely, because male mating investment strongly influences the reproductive outcome of individual matings [11], [22], [33], [34], [35], male investment will influence not only male, but also female fitness. Thus, changes in mating investment by both males and females as a consequence of their age can influence age-specific fitness (and thus, age-specific reproductive value) of both sexes. Although many studies have examined the effects of either male or female age on mating effort [9], [14], [15], [31], [33], [36], [37], the effects of interactions between male and female age on mating effort––particularly that of males––and on fitness are largely unexplored. Yet, evaluating the combined effects of male and female age on male mating investment is important, in order to ascertain how age-specific investment in reproduction influences fitness and the evolution of reproductive traits in age-structured populations.

Our goal was to evaluate how males alter mating investment as a function of both their own age and their mate’s age. We also evaluated the fitness effects of male investment by assaying female fecundity in response to male investment. As we describe below, we used *Drosophila pseudoobscura* to study the combined effects of male and female age on copulation duration. Copulation duration is often used as a measure of male investment in mating [9], [11], [34], [38], [39], [40], because it is associated with the size of male ejaculate transferred during mating in a wide variety of insect taxa [16], [41]. We further assayed the fitness effects of copulation duration by measuring the number of eggs females laid following mating.

## Methods

### Experimental Population

The flies for this study came from a laboratory population of *D. pseudoobscura* that was founded in August 2009 with wild-caught flies (10 females and 15 males) collected from Flagstaff, Arizona, USA. All necessary permissions for collection activities were obtained from Flagstaff City Parks department. The study species is not an endangered or protected species, and no additional permissions were required for collection. To establish the population, we mated progeny from all the wild females in a complete reciprocal cross design (100 mating combinations with three to six replicates). Some of the offspring of each wild female were also mated to at least three of the fifteen wild males. The resulting flies from these matings were then mixed to establish a thoroughly mixed population. This population was maintained with overlapping generations on a cornmeal agar medium under a 12∶12 light-dark cycle at 19°C and 60–80% relative humidity for five and a half months before starting the experiments described below.

To initiate our experiments, we randomly selected 50 flies (25 males and 25 females) from the stock population. These randomly chosen flies were allowed to mate and oviposit in 170 ml stock bottles for 6 days. Male and female virgin flies were obtained from the offspring produced in these bottles. We isolated virgin flies within 8 hours from eclosion and housed them individually in 50 ml vials, which contained cornmeal agar medium supplemented with yeast granules. Each day, these isolated virgin flies were randomly allocated to the different experimental age treatments so as to avoid any effects of eclosion date.

### Mating Trials

To evaluate the effects of male and female age on male investment, we used mating trials in which we paired males of different ages with females of different ages and measured copulation duration.

We used copulation duration as our measure of male mating investment, because it is generally correlated with the amount of sperm and/or other components of seminal fluid transferred during mating [9], [33], [42], [43], [44]. In *D. melanogaster*, for example, copulation duration is associated with the transfer of seminal fluid proteins (Sfps) [44], which can have a large effect on male and female fitness [45], [46], [47]. Indeed, a number of Sfps play a key role in sperm competition [46], [48], [49]. Because copulation duration has these fitness enhancing effects, it is often used as a measure of male investment in individual matings [9], [11], [29], [34], [40].

To determine the effect of male and female age on copulation duration, virgin females aged 4 days or 11 days were mated to 4-, 8-, 11-, 15- or 19-day old virgin males. We chose these age treatments because they likely represent age classes that are most commonly present in natural populations of *D. pseudoobscura* [33], [50]. We did not include older flies, because an estimated 70% of flies die within 14 days from eclosion in the wild [50]. Male *D. pseudoobscura* usually attain reproductive maturity within 12 hours of eclosion, and carry a full complement of sperm at the age of 2 days [51]. Thus our age classes represented those of sexually mature males that would most likely be exposed to selection in natural populations.

For each mating trial, a single female from one of the two female age treatments was randomly paired with a single male from one of the five male age treatments. To do so, a male was aspirated into the female’s vial and he was allowed to mate. In addition to copulation duration, we measured mating latency as the time between introduction of the male into the vial and the start of copulation. We used this mating latency as a measure of female mate preference. Each trial ended when copulation stopped or at 10 minutes if the pair failed to initiate copulation. Virgin *D. pseudoobscura* mate readily, and only two of our pairings failed to initiate copulation within 10 minutes. We conducted all trials between 2 hours and 6 hours after the lights came on in the incubator. We randomized the order of mating trials on a given day with respect to male and female ages to avoid any order effects, and an equal number of mating trials were conducted on each day for all male-female age combinations. All trials took place in an observation room at 22–25°C with 50–70% relative humidity. We excluded any matings that were shorter than 60 seconds, because these are likely to be pseudocopulations [52], [53]. This resulted in the following sample sizes for the female-male age combinations: f4-m4∶16, f4-m8∶11, f4-m11∶8, f4-m15∶10, f4-m19∶10, f11-m4∶10, f11-m8∶10, f11-m11∶10, f11-m15∶7, f11-m19∶7.

In natural populations, older males are unlikely to be virgins. To determine whether any difference in the behavior of older males was a result of age itself or of virginity at old age, we mated a set of 10-day old males to 4-day old virgin females (N = 7). The females were discarded, while the males were retained for remating. When these males were 11 days old, they were then mated again to 4-day old females, and the duration of copulation during these matings was measured.

Unfortunately, an incubator failure that resulted in the loss of the study population prevented higher sample sizes in the experiment. Nevertheless, the effects of interest in this study were large enough to be detected with our sample sizes (see Results).

### Early Post-mating Fecundity

Following the mating trials, we measured the effect of copulation duration and male and female age on early post-mating fecundity. To do so, young and old females that had been mated to the youngest, 4-day old, males (N young females = 15, N old females = 9), intermediate-aged, 11-day old, males (N young females = 7, N old females = 10), and the oldest, 19-day old, males (N young females = 8, N old females = 6) were allowed to oviposit on grape juice-agar medium, supplemented with yeast granules, for two days post copulation. Grape juice-agar medium provided better egg visibility for counting. Because virgin females potentially lay a small number of unfertilized eggs, we collected all eggs and maintained them for 10 days at 19°C to confirm that they were fertilized (most eggs hatch within three days at 19°C). Three matings resulted in unfertilized eggs (all three matings were between 4-day old males and 4-day old females). These three matings were excluded from analysis because male fertility could not be ascertained.

Females that did not lay any eggs during the two-day period were monitored for an additional 8 days to determine whether they could produce eggs. Two females failed to lay any eggs during these additional 8 days and were therefore removed from subsequent analyses of fecundity as they were likely infertile. Their removal did not affect the outcome of the analyses. Three females were lost during handling and their fecundity could not be measured.

### Statistical Analysis

All statistical analyses were performed in R ver. 2.15.1 [54]. The effects of male and female age on mating latency and copulation duration were analyzed using generalized linear models (glm) and a type III ANOVA. Because the copulation duration data showed a quadratic relationship between the variance and the mean (see Fig. S1 in file S1; Table S1 in file S1), a Gamma distribution (with log link function) was used in these analyses. The log link function eliminates meaningless negative time estimates for copulation duration. Male age and female age were modeled as discrete variables because we used only certain age classes.

The mean-variance relationship of egg laying data grouped by different male-female age combinations revealed that the data were overdispersed compared to a Poisson distribution (Table S2 in file S1). We therefore used the GAMLSS package (Generalized Additive Models for Location, Scale and Shape) in R [55] to analyze the relationship of early post-mating fecundity with copulation duration, male age and female age. Unlike glm models, gamlss models enable likelihood-based analysis of over-dispersed data. This allowed us to determine which predictor variables and which interactions, if any, generated a model that best fits the data. These log-likelihood tests showed that a negative binomial distribution with a linear mean-variance relationship fit the data better than a Poisson distribution (see Table S3 in file S1). We fit a set of gamlss models in which we evaluated the individual and combined effects of copulation duration, male age and female age on mean early post-mating fecundity. We then used the Akaike Information Criterion with a correction for small sample sizes (AICc) to select the model with the combination of our predictor variables that best explained the variation in early post-mating fecundity of females [56]. We note that AICc is an approximate correction for models with non-normal error distribution.

All raw data for copulation duration, latency, egg laying rates and male remating can be found in the files S2, S3, S4 and S5, respectively.

## Results

### Mating Trials

In our mating trials, we did not find any significant effect of male or female age on latency to mate (Generalized Linear Model, male age:- t = 1.52, p = 0.13; female age:- t = 1.24, p = 0.22). Although there was variation in the time it took for males to encounter the females, all males initiated courtship immediately on encountering the female. All females mated within less than 3 seconds after initiation of courtship by any male. Such behavior of virgin females is normal and has been observed in other populations of *D. pseudoobscura* [57], (M. Noor, personal communication).

**Figure 1:**
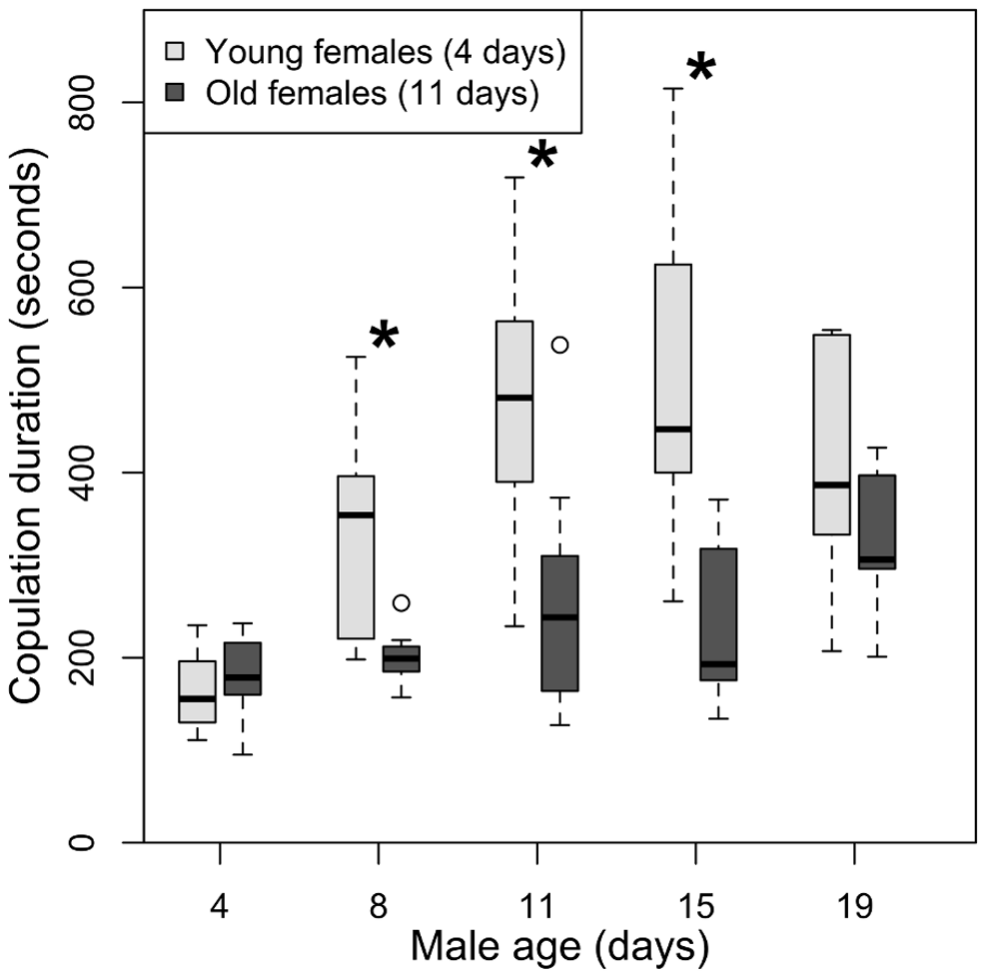
The effect of male age on copulation duration with young females (light grey boxes) and old females (dark grey boxes). Asterisks indicate significant differences in copulation duration between old and young females within a male age class.

By contrast, copulation duration increased with male age regardless of whether males were mated to young or old females (Fig. 1; ANOVA of glm, p<0.0001; see file S1 for detailed model results). The effect of female age was only observed for intermediate-aged males. In particular, 8-day, 11-day and 15-day old males mated for a significantly longer duration with young females than with old females (Fig. 1; Generalized Linear Model; t-values, 8-day: −3.09, 11-day: −3.29, 15-day: −3.80; all p values <0.003). Consequently, the increase in copulation duration with male age was more pronounced in matings with younger females than with older females (ANOVA of glm with interaction, p<0.001).

Contrary to the findings with the intermediate aged males, copulation duration for the youngest (4-day old) and the oldest (19-day old) males did not differ between females of different ages (Fig. 1; Generalized Linear Model, 4-day old males: t = 0.42, p = 0.67; 19-day old males: t = −1.28, p = 0.20).

Copulations of 11-day old males that were previously mated to young females at the age of 10 days (mean = 490 seconds) were significantly longer than the virgin 4-day old males (mean = 165.5 seconds; glm, t = 8.857, p<0.01; Fig. S2 in file S1), but not different from virgin 11-day old males (mean = 477.8 seconds; Generalized Linear Model, t = 0.184, p = 0.855; Fig. S2 in file S1).

### Early Post-mating Fecundity

We used the AICc and log-likelihood ratios to identify the statistical model that best explained variation in early post-mating fecundity (see file S1 for details of model selection and AICc tables). The best fitting model included effects of copulation duration and female age on early post-mating fecundity. A second model with a marginally higher AICc value (but lower AIC value; see Table S4 in file S1) also included significant effects of male age and an interaction between male age and copulation duration on early post-mating fecundity. Given the similarity in AICc values of these two models, and the approximate nature of the AICc correction for models with non-normal errors, we discuss the results of both models. For results that are qualitatively identical for both models, statistics of only the best model are reported here. Detailed output of both models is presented in the file S1.

**Figure 2:**
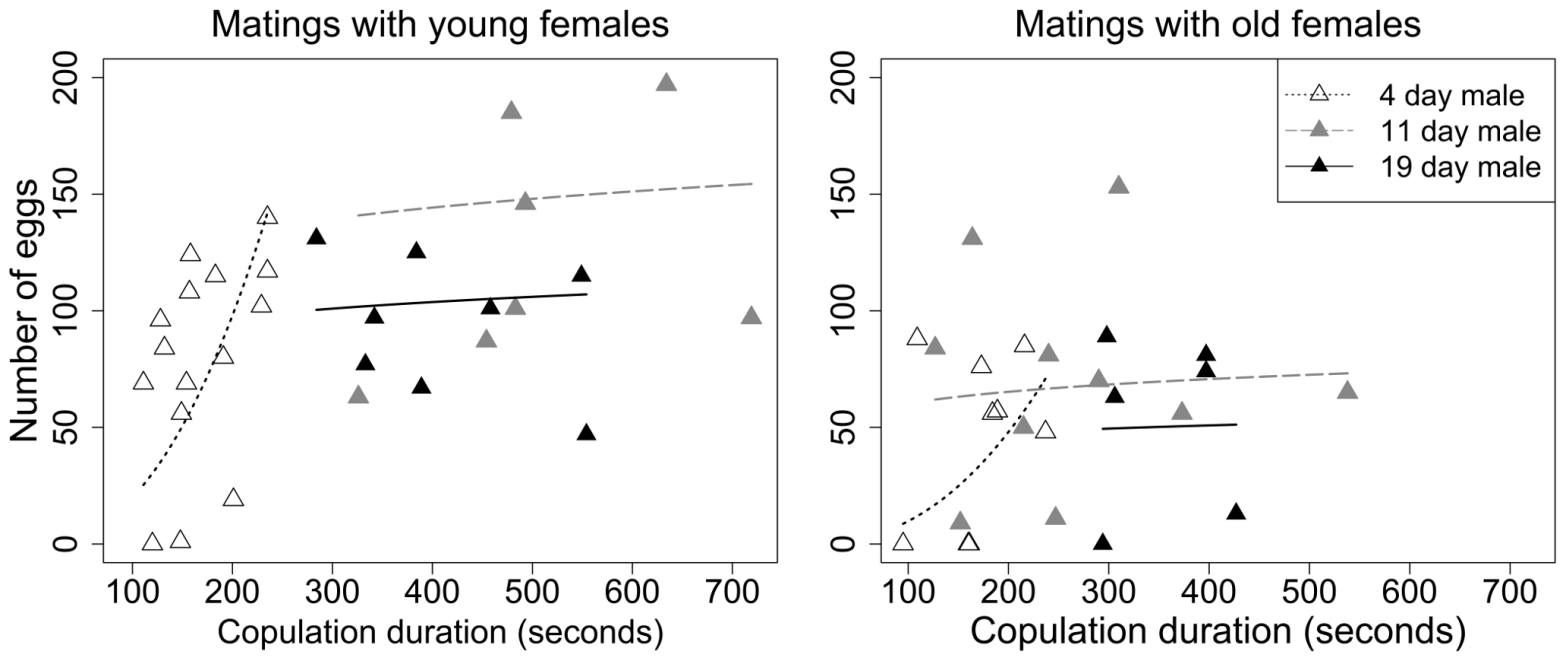
Effect of copulation duration on the number of eggs laid by females in the first two days after mating. Regression lines obtained from the second gamlss models are plotted. Empty symbols and dashed lines represent matings of 4-day old males; grey symbols and solid grey line depict matings of 11-day old males; filled black symbols and solid black line show matings of 19-day old males.

We found that female age significantly affected the number of eggs laid within two days after copulation: young females laid more eggs than old females (Fig. 2; gamlss, t = −2.57, p = 0.013). We also found that longer copulations resulted in a higher number of eggs laid by the females (gamlss, t = 3.57, p<0.01).

According to the second model, male age also affected the number of eggs produced such that females tended to lay more eggs when mated to older males. This pattern was most pronounced for females mated to 11-day old males (who have the longest copulations) compared to those mated to 4-day old males (who have the shortest copulations; second gamlss model, t = 2.86, p = 0.006). Females mated to 19-day old males also tended to produce more eggs than females mated to 4-day old males, but the difference was marginal (second gamlss model, t = 1.97, p = 0.055).

Moreover, according to the second model, a significant interaction exists between the effects of copulation duration and male age on early post-mating fecundity (second gamlss model, 4-day old males vs. 11-day old males: t = −2.81, p = 0.007; 4-day old males vs. 19-day old males: t = −2.10, p = 0.041). Copulation duration was positively correlated with the number of eggs laid by females mated to 4-day old males. By contrast, copulation duration did not predict the number of eggs laid by females that had been mated to 11-day and 19-day old males (Fig. 2).

Aside from the uncertainty as to which model best explains early post-mating fecundity, this interaction between male age and copulation duration should be interpreted with caution for two additional reasons. First, the lack of a relationship between copulation duration and the number of eggs produced by females mated to 11- and 19-day old males may be an effect of the small sample size. Second, copulation duration by the youngest males did not overlap with copulation duration by the intermediate-aged or oldest males. Thus, copulation duration and male age are confounded in these groups (see [33] for a similar effect of male age on copulation duration).

## Discussion

We evaluated how male and female age interact to affect copulation duration (our measure of male mating investment) and female fecundity immediately after mating. We found that investment in mating generally increases with male age, but that intermediate-aged males make the largest investment in mating. We also found that male *D. pseudoobscura* discriminate between females of different ages, and invest more in matings with young females. Moreover, this pattern of mate discrimination coincided with the pattern of mating investment, such that intermediate-aged males displayed the largest difference in investment between young and old females (Fig. 1).

Why should older males copulate for longer than younger males? One explanation is that greater investment in copulation duration by older males represents an evolved response to decreasing chances of future reproduction as they age [23], [24], [25], [26], [27], [28]. This hypothesis posits that older males will be selectively favored to invest maximally in current matings, because they are less likely to mate in the future. Thus, as males age and opportunities for future reproduction diminish, males should increase investment in any given reproductive bout. Our data were consistent with this prediction. An alternative possibility is that lengthy copulations of older males result from physiological degradation, such that older males require more time to transfer the same amount of resources compared to younger males. However, Avent et al. [33] found that older *D. pseudoobscura* males transfer higher amounts of sperm during their long matings. Thus, physiological degradation is unlikely to explain our results. We also find that even previously mated intermediate-age males copulate for longer than the youngest males. This result indicates that the increase in copulation duration with age observed in the virgin males cannot be explained solely by the virgin status of 11-day old males, and is instead an effect of male age.

Moreover, male *D. pseudoobscura* increase mating investment when exposed to competitors [58]. Thus, the pattern of increasing mating investment with age may become even more exaggerated in natural populations, because older males are more likely to have been exposed to competitors in nature than younger males.

Another implication of the decreasing opportunities of mating with age is that males should become less discriminatory as they age. As males age, any mating opportunity becomes increasingly likely to be their last. Therefore, old males should invest whatever resources they have in whichever mating opportunity they can find. Thus, although males are expected to invest more in a given mating as they get age, males are also expected to be less discriminatory in their choice of mate with which they make that investment.

Our findings provided mixed support for the notion that males should become less discriminatory with age. At the oldest age class, 19 days, males invested equally in old and young females. That is, copulation duration by males did not differ between old and young females. Because only about 20% of flies are predicted to survive for 19 days in wild populations [50], 19-day old males would have very low chances of future matings in the wild. Thus, making a high investment in a mating, regardless of the mate’s age, will potentially be selectively favored.

However, contrary to the above hypothesis that males should become less discriminatory as they age, we found that the youngest males did not differ in copulation duration with old versus young females. Indeed, males appeared to become increasingly discriminatory until the last age class (Fig. 1): the difference in copulation duration with old versus young females was most pronounced for intermediate-aged males (Fig. 1). This pattern suggests that as reproductive investment increased, so too did discrimination up to the oldest age class.

Resource limitation might explain, in part, both the lower overall investment by younger males, and their apparent lack of discrimination among different females. In *D. pseudoobscura*, males do not even begin sperm transfer until about 90 seconds after copulation is initiated [59]. The average copulation duration for young males was 169 seconds, which is closer to this minimum time than the average copulation duration for older males with young females (8-day: 336.7 s; 11-day: 477.8 s; 15-day: 486.5 s; 19-day: 405.5 s). If young males have not accumulated sufficient resources, they may be restricted to investing in the minimum time required for sperm transfer. By contrast, males aged between 8 and 15 days may have acquired more resources over their lifetime and are therefore able to both engage in longer copulations and differentially invest in copulation duration depending on the female.

Because older females laid fewer eggs than younger females (Fig. 2), males would be expected to invest more in matings with younger females. Our finding that intermediate-aged (8–15-day old) males appeared to be the most discriminating may reflect access to sufficient resources, on the one hand, and sufficient opportunities for future matings on the other hand. By contrast, the youngest males might have inadequate resources to make such differential allocations, whereas the oldest males might be under selection to make maximal investments into a mating regardless of the female because their opportunities for future matings are so low. Generally, males should be most discriminating at intermediate ages when they have both resources *and* opportunities for future reproduction.

From the female’s perspective, we found that females laid more eggs following longer copulations. Consequently, females mated to intermediate-aged males laid more eggs than those mated to the youngest or the oldest males. Jones and Elgar [37] have also found a similar pattern in hide beetles (*Dermestes maculatus*). One possible explanation for this is that females adjust their own reproductive investment depending on either their mate’s age or their mate’s investment in the mating. However, even *within* the matings with the youngest males females showed higher production of eggs in response to longer copulation times, indicating that the females were not responding solely to male age per se. Thus, females may invest more in matings in which the male also provides enhanced investment.

Moreover, longer copulations may result in the transfer of larger amounts of seminal fluid proteins that stimulate early post-mating fecundity, as occurs in *D. melanogaster* [44]. In the absence of direct measures of seminal fluid proteins transferred by the different males, we cannot ascertain whether this possibility accounts for our results. However, given previous findings from *D. melanogaster* [44], [60], our results are consistent with this possibility.

The second best statistical model suggests that although females produced more eggs in response to longer copulations with 4-day old males, such a pattern did not arise for the older males (11- and 19-day old). Because there is little to no overlap in duration of copulation by the youngest (4-day old) males and the older (11- and 19-day old) males, this result is difficult to interpret. The result may suggest that copulation duration of older males does not correlate with transfer of seminal fluid proteins that influence egg-laying rates of females. Alternatively, such a pattern could arise if females are unable to increase egg-laying beyond certain physiological limits despite increasing male copulation times. An experimental resolution between these explanations will require artificial interruption of copulation duration of old males. Such manipulations are associated with their own complications [42] and are beyond the scope of this study. Interestingly, in a study on female mating preferences by Avent et al. [33], young female *D. pseudoobscura* that were mated with old (14-day old) males did not differ from those mated with young (2-day old) males in early post-mating fecundity, but did exhibit higher late post-mating fecundity. The discrepancy between these findings and our results in this study may be a result of differences between populations and between experimental protocols. The population used by Avent et al. had been maintained in the lab environment for 12 to 17 months, and most likely was exposed to strong selection due to high sperm competition (TAR Price, personal communication). Furthermore, males in the experiment by Avent et al. were exposed to competitors prior to mating. Such selection and exposure to competitors may explain the longer copulation durations of their males than males of similar ages in this study. If maximal fecundity stimulation results from high mating investment in all matings, the effects of male investment on early post-mating fecundity may not become apparent.

Our study highlights the way in which differential reproductive investment by males and females can interact to affect the dynamics of both mate discrimination and age-specific fitness from a given mating. These dynamics can ultimately affect the strength and mode of selection in age-structure populations [28]. Indeed, age-dependent changes in mating investment will also likely alter the dynamics of sperm competition, and competition itself can alter male mating investment [7], [22]. Further empirical and theoretical work is needed to better understand these interactions and their ultimate effects on sexual selection and differentiation among populations that might differ in resource availability, age structure, survivorship, and mate competition.

## Supporting Information

File S1 **Details of statistical analyses are reported including supplementary figures and tables.** Also included are the results for male remating trials.
(PDF)

File S2 **A.csv format file with all the data used for the analysis of the effects of male age on copulation duration is included.**
(CSV)

File S3 **A.csv format file with all the data used for the analysis of mating latency is included.**
(CSV)

File S4 **A.csv format file with all the data used for the analysis of egg laying rates is included.**
(CSV)

File S5 **A.csv format file with all the data used for the analysis of the effects of male mating status on copulation duration is included.** Male mating status is indicated as ‘V’ for virgin males and as ‘NV’ for non-virgin males.
(CSV)
