# Supplementary material for: Age-Dependent Male Mating Investment in Drosophila pseudoobscura
Source: PLoS One. 2014 Feb 19;9(2):e88700. doi: 10.1371/journal.pone.0088700 (PMC3929311; doi:10.1371/journal.pone.0088700)
Supplement: File S1 — Details of statistical analyses are reported including supplementary figures and tables. Also included are the results for male remating trials. (PDF) [file pone.0088700.s001.pdf]

Supplementary Material

Age-dependent male mating investment in *Drosophila pseudoobscura*

Sumit Dhole and Karin S. Pfennig

I. Copulation Duration

Table S1: The copulation duration data are overdispersed relative to a Poisson distribution (variance to mean ratio > 1).

Female age	Male age	Mean	Variance	Variance to mean ratio
4	4	165.5	1778.0	10.7
4	8	336.7	13044.6	38.7
4	11	477.8	23697.6	49.6
4	15	486.5	27790.3	57.1
4	19	405.4	14626.7	36.1
11	4	174.6	2132.3	12.2
11	8	199.9	793.2	4.0
11	11	265.6	14922.5	56.2
11	15	240.6	9260.3	38.5
11	19	331.4	6318.3	19.1

Figure S1: Linear relationship between the mean and the standard deviation (quadratic relationship with variance) in copulation duration measured in the different male-female age combination groups.

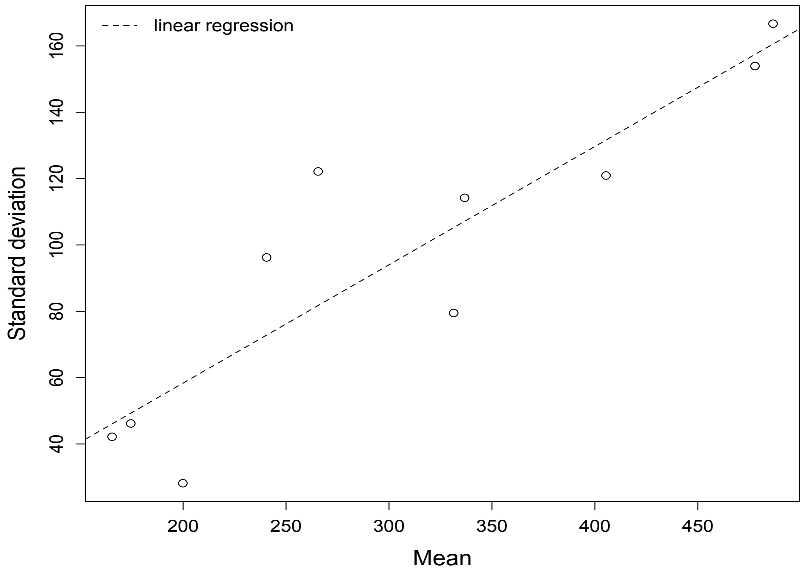

Output for the generalized linear model:

Call:
glm(formula = Copulation.duration.seconds ~ factor(Male.age) * factor(Female.age), family = Gamma(link = log), data = copudata)

Deviance Residuals:

Min	1Q	Median	3Q	Max
-0.65722	-0.21524	-0.04602	0.17905	0.79967

Coefficients:

	Estimate	Std. Error	t value	Pr(>|t|)
(Intercept)	5.10897	0.07828	65.266	< 2e-16 ***
factor(Male.age)8	0.71030	0.12264	5.792	1.03e-07 ***
factor(Male.age)11	1.06012	0.13558	7.819	1.02e-11 ***
factor(Male.age)15	1.07827	0.12622	8.543	3.32e-13 ***
factor(Male.age)19	0.89590	0.12622	7.098	2.95e-10 ***
factor(Female.age)11	0.05353	0.12622	0.424	0.672541
factor(Male.age)8:factor(Female.age)11	-0.57498	0.18614	-3.089	0.002680 **
factor(Male.age)11:factor(Female.age)11	-0.64062	0.19491	-3.287	0.001452 **
factor(Male.age)15:factor(Female.age)11	-0.75775	0.19935	-3.801	0.000263 ***
factor(Male.age)19:factor(Female.age)11	-0.25499	0.19935	-1.279	0.204198

Signif. codes: 0 ‘***’ 0.001 ‘**’ 0.01 ‘*’ 0.05 ‘.’ 0.1 ‘ ’ 1

(Dispersion parameter for Gamma family taken to be 0.0980428)

Null deviance: 23.975 on 98 degrees of freedom
Residual deviance: 8.892 on 89 degrees of freedom
AIC: 1169.9

Number of Fisher Scoring iterations: 4

Confidence intervals:

	Estimate	2.5 %	97.5 %
(Intercept)	5.10897	4.9593716	5.2664281
factor(Male.age)8	0.71030	0.4714257	0.9527546
factor(Male.age)11	1.06012	0.7979036	1.3301991
factor(Male.age)15	1.07827	0.8329134	1.3283389
factor(Male.age)19	0.89590	0.6505508	1.1459762
factor(Female.age)11	0.05353	-0.1918258	0.3035996
factor(Male.age)8:factor(Female.age)11	-0.57498	-0.9408627	-0.2106510
factor(Male.age)11:factor(Female.age)11	-0.64062	-1.0248667	-0.2601819
factor(Male.age)15:factor(Female.age)11	-0.75775	-1.1481843	-0.3659588
factor(Male.age)19:factor(Female.age)11	-0.75775	-0.6454264	0.1367991

Type III anova of the above glm model:

Analysis of Deviance Table (Type III tests)

Response: Copulation.duration.seconds

	LR Chisq	Df	Pr(>Chisq)
factor(Male.age)	96.010	4	< 2.2e-16 ***
factor(Female.age)	0.181	1	0.6708971
factor(Male.age):factor(Female.age)	20.663	4	0.0003693 ***

Signif. codes: 0 '***' 0.001 '**' 0.01 '*' 0.05 '.' 0.1 ' ' 1

Figure S2: Copulation duration of virgin (V) and non-virgin (NV) males of two ages. Eleven-day old males copulate for longer than young 4-day old males regardless of mating status.

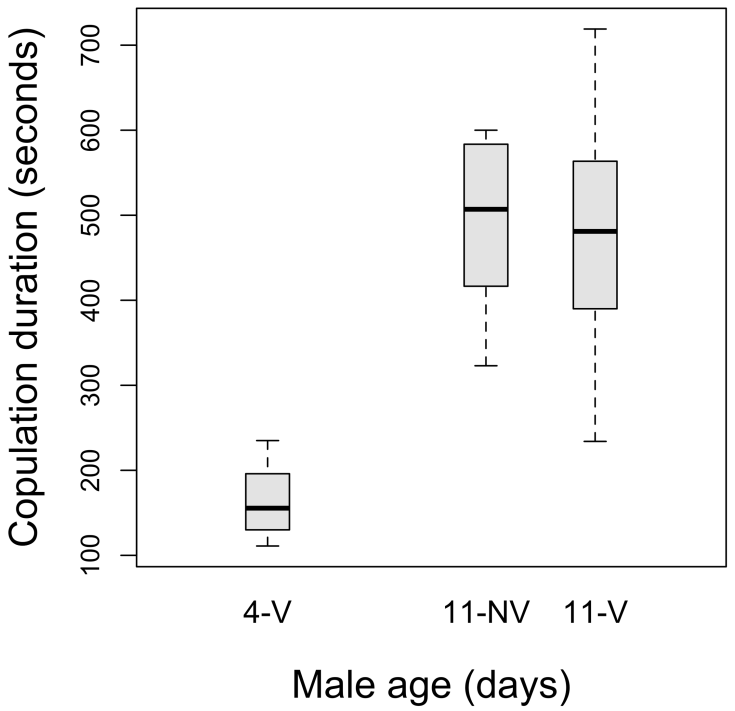

II. Early post-mating fecundity

Table S2: Mean-variance relationship of egg count data for all male-female mating combinations

Female age	Male age	Mean	Variance	Variance to mean ratio
4	4	78.7	1916.0	24.4
4	11	125.1	2645.5	21.1
4	19	95.0	864.0	9.1
11	4	45.6	1344.5	29.5
11	11	71.0	2082.2	29.3
11	19	53.3	1405.9	26.4

The variance to mean ratios for all the groups fall within a small range, with the exception of 4-day old females mated with 19-day old males, suggesting a linear mean-variance relationship. Models with a binomial distribution with a linear mean-variance relationship ('NB2' distribution in the GAMLSS package) were found to fit the data better than corresponding models with a negative binomial distribution with a quadratic mean-variance relationship ('NB1' distribution) or a Poisson distribution. NB1 vs NB2 vs Poisson comparisons are shown for the three best fitting models in Table S3. Results for all NB2 models are shown in Table S4.

Table S3: AICc values for the best three models with NB1, NB2 and Poisson error distributions

Distr.	Model	Predictors	K	AICc
**NB2**	Sans male age, sans interaction	ln(Copulation duration)+ Female age	4	576.9
**NB1**	Sans male age, sans interaction	ln(Copulation duration)+ Female age	4	592.4
**Poisson**	Sans male age, sans interaction	ln(Copulation duration)+ Female age	4	1741.5
**NB2**	One two-factor interactions	ln(Copulation duration)* Male age + Female age	8	577.1
**NB1**	One two-factor interactions	ln(Copulation duration)* Male age + Female age	8	600.8
**Poisson**	One two-factor interactions	ln(Copulation duration)* Male age + Female age	8	1849.8
**NB2**	Sans male age with interaction	ln(Copulation duration)* Female age	5	579.1
**NB1**	Sans male age with interaction	ln(Copulation duration)* Female age	5	594.8
**Poisson**	Sans male age with interaction	ln(Copulation duration)* Female age	5	1848.2

The small difference between AICc values of the two best models (Table S4) and their low Akaike weights suggest uncertainty in exclusion of the effect of male age. However, the top four best fitting models consistently include a significant independent effect of copulation duration on early post-mating fecundity.

It should be noted that the AICc correction is designed for models with Gaussian error distribution, and the correction does not generalize in a straightforward way to models with non-Gaussian distributions. Correction for models with negative binomial distribution is not available. However, this correction is argued to be better than no correction for small sample sizes (Simonoff 2003).

Table S4: Akaike Information Criterion indices (AIC), Akaike Information Criterion indices corrected for small sample size (AICc), the number of parameters (K), Log Likelihood statistics (LL) and the Akaike weights (w) of different models are listed. The models are arranged by their AICc values.

No.	Model	Predictors	K	LL	AIC	AICc	w
**1**	Sans male age, sans interaction	ln(Copulation duration)+ Female age	4	–284.1	576.1	576.9	32.8%
**2**	One two-factor interactions	ln(Copulation duration)* Male age + Female age	8	–279.0	574.0	577.1	29.8%
**3**	Sans male age with interaction	ln(Copulation duration)* Female age	5	–283.9	577.8	579.1	11.1%
**4**	Two two-factor interactions	ln(Copulation duration)* Male age + ln(Copulation duration)* Female age	9	–278.7	575.5	579.5	9.2%
**5**	Main effects only	ln(Copulation duration) + Male age + Female age	6	–283.1	578.1	579.9	7.4%
**6**	Sans copulation duration	Male age+ Female age	5	–284.5	579.0	580.2	6.4%
**7**	One two-factor interactions	ln(Copulation duration)* Female age + Male age	7	–282.8	579.6	582.0	2.6%
**8**	Three two-factor interactions	ln(Copulation duration)* Male age + ln(Copulation duration)* Female age + Male age*Female age	11	–278.3	578.5	584.7	0.7%
**9**	Female age only	Female age	3	–290.2	586.4	586.8	0.2%
**10**	3-factor interaction	ln(Copulation duration)* Male age* Female age	13	–277.1	580.3	589.1	0.1%

A summary of the two best models is shown below.
(Note: In gamlss models, “log” refers to the natural log.)

Best model:

```
*******************************************************************
Family:  c("NBII", "Negative Binomial type II")

Call:  gamlss(formula = Eggs ~ log(Copulation.duration.seconds) + factor(Female.age),
    family = NBII, data = eggdata)

Fitting method: RS()
```

Mu link function: log
Mu Coefficients:

	Estimate	Std. Error	t value	Pr(>|t|)
(Intercept)	0.9157	1.0496	0.8725	0.3870502
log(Copulation.duration.seconds)	0.6459	0.1794	3.5993	0.0007213
factor(Female.age)11	-0.5451	0.2113	-2.5801	0.0127990

Sigma link function: log
Sigma Coefficients:

Estimate	Std. Error	t value	Pr(>|t|)
3.908e+00	2.291e-01	1.706e+01	1.018e-22

No. of observations in the fit: 55
Degrees of Freedom for the fit: 4
Residual Deg. of Freedom: 51
at cycle: 5

Global Deviance: 568.1132
AIC: 576.1132
SBC: 584.1425
****

Confidence intervals:

	Estimate	2.5 %	97.5 %
(Intercept)	0.9157	-1.1571737	2.9886597
log(Copulation.duration.seconds)	0.6459	0.2909656	1.0008074
factor(Female.age)11	-0.54511	-0.9606749	-0.1295458

Second best model:
****
Family: c("NBII", "Negative Binomial type II")
Call: gamlss(formula = Eggs ~ log(Copulation.duration.seconds) * factor(Male.age) + factor(Female.age), family = NBII)

Fitting method: RS()

Mu link function: log
Mu Coefficients:

	Estimate	Std. Error	t value	Pr(>|t|)
(Intercept)	-7.589	3.6375	-2.086	0.042390
log(Copulation.duration.seconds)	2.298	0.6944	3.309	0.001804*
factor(Male.age)11	11.868	4.1556	2.856	0.006370*
factor(Male.age)19	11.660	5.9299	1.966	0.055176
factor(Female.age)11	-0.713	0.2289	-3.114	0.003137*
log(Copulation.duration.seconds):factor(Male.age)11	-2.182	0.7758	-2.813	0.007146*
log(Copulation.duration.seconds):factor(Male.age)19	-2.202	1.0505	-2.097	0.041436*

Sigma link function: log
Sigma Coefficients:

Estimate	Std. Error	t value	Pr(>|t|)
3.752e+00	2.283e-01	1.643e+01	4.128e-21

No. of observations in the fit: 55
Degrees of Freedom for the fit: 8
Residual Deg. of Freedom: 47
at cycle: 4

Global Deviance: 557.9931
AIC: 573.9931
SBC: 590.0518
****

Confidence intervals:

	Estimate	2.5 %	97.5 %
(Intercept)	-7.589	-14.70528089	-0.4735016
log(Copulation.duration.seconds)	2.298	0.93725102	3.6582401
factor(Male.age)11	11.868	3.78774969	19.9482951
factor(Male.age)19	11.660	-0.04658854	23.3673123
factor(Female.age)11	-0.713	-1.16331291	-0.2626987
log(Copulation.duration.seconds):factor(Male.age)11	-2.182	-3.68557690	-0.6786359
log(Copulation.duration.seconds):factor(Male.age)19	-2.202	-4.26885266	-0.1360741

References:

Simonoff JS (2003) Analyzing Categorical Data. New York: Springer-Verlag.
